# *Staphylococcus aureus* bacteremia complicated with non-traumatic mediastinal abscess in children: A case report

**DOI:** 10.3389/fped.2023.1115788

**Published:** 2023-02-28

**Authors:** Yiyuan Li, Yu Zhu, Chaomin Wan, Yang Wen

**Affiliations:** Key Laboratory of Women and Children Diseases, Department of Pediatrics, West China Second University Hospital, Sichuan University, Laboratory of Birth Defects and Related Diseases of Women and Children (Sichuan University), Ministry of Education, Chengdu, China

**Keywords:** *Staphylococcus aureus* bacteremia, non-traumatic mediastinal abscess, pediatric, healthy children, bacteria with unknown origin

## Abstract

**Background:**

*Staphylococcus aureus* bacteremia complicated with non-traumatic mediastinal abscess rarely occurs in children. Herein, we report a case of S. aureus bacteremia in a previously healthy 15-month-old boy, which was complicated with a non-traumatic mediastinal abscess, followed by recovery without surgery

**Case presentation:**

A previously healthy 15-month-old boy presented to the hospital with a high fever, accompanied by chills, lethargy, tachycardia, tachypnea, and slight cough. Contrast-enhanced computerized tomography revealed mediastinal abscess and blood culture analysis showed the presence of *S. aureus* which was methicillin-susceptible. With prompt initiation of antibiotic treatment, with appropriate duration, the patient successfully recovered without surgical drainage upon discharge.

**Conclusions:**

*Staphylococcus aureus* bacteremia complicated with non-traumatic mediastinal abscess is rare in children, and early recognition and appropriate management are essential for a successful outcome.

## Background

*Staphylococcus aureus* is a major pathogen responsible for invasive infections such as bacteremia, endocarditis, osteomyelitis, arthritis, and pneumonia (1). *Staphylococcus aureus* bacteremia (SAB) is associated with high mortality and morbidity despite the availability of adequate treatments (2, 3). Intravascular catheters, implants, chronic diseases, nasal colonization, and intravenous drug use are the main risk factors for SAB (2, 4–6). Patients with SAB frequently experience high fever, sepsis, toxic shock syndrome, and other symptoms associated with the source of infection, including osteomyelitis, arthritis, endocarditis, and pneumonia. The most common complications of SAB are necrotizing pneumonia, pulmonary bullae, pleural effusion, and empyema; however, mediastinal abscess (MA) is relatively rare in children with SAB. MA is rarely observed in healthy children, and usually occurs as a complication of esophageal perforation, thoracic trauma, or thoracic surgery (7, 8). MA due to a non-traumatic etiology is extremely infrequent in pediatric population and tends to result either from direct extension along contiguous anatomic pathways and fascial planes or by hematogenous or lymphatic spread from distant sites of infection (9, 10). In this report, we describe a case of a 15-month-old boy who presented with SAB further complicated with non-traumatic MA. The patient had a successful recovery after prompt antibiotic therapy, without resorting to surgical drainage.

## Case presentation

A 15-month-old boy was admitted to the hospital because of prolonged and persistent fever for fifteen days. The patient was delivered at full term weighing 3,100 g and did not require admission to the neonatal intensive care unit (NICU). He was previously healthy without recurrent respiratory infections, cough, wounds, admissions, or surgeries. Additionally, he was up-to-date with vaccinations.

**Figure 1:**
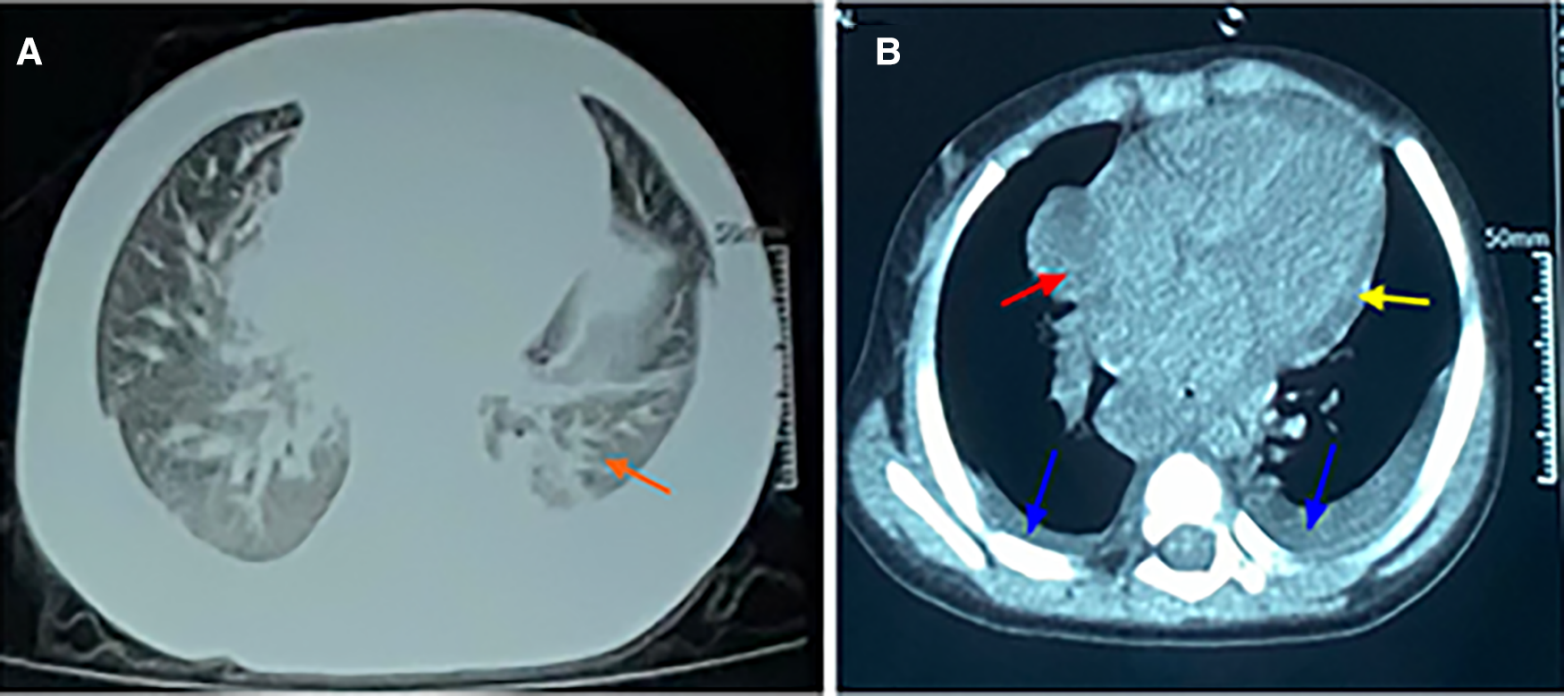
CT scan of chest. (**A**) The lung window showing scattered infiltrates in the bilateral lungs. (**B**) The mediastinal window showing an enlarged mediastinum (red arrow), some pleural effusion (blue arrow), and pericardial effusion (yellow arrow).

**Figure 2:**
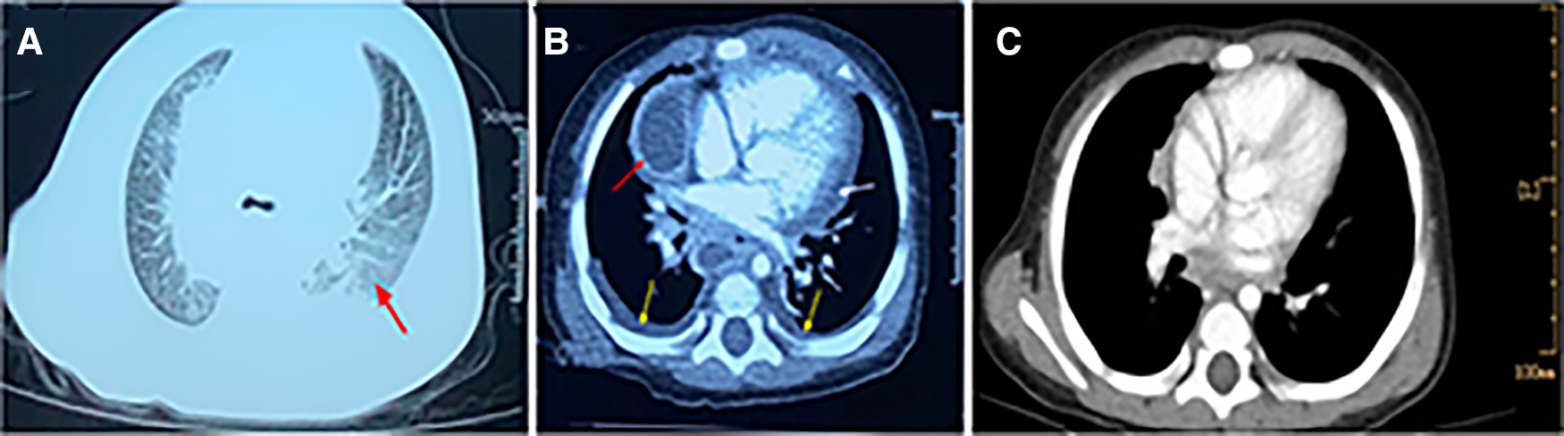
Contrast-enhanced CT scan of chest. (**A**). CT showing scattered infiltrates in the bilateral lungs. (**B**). CT showing partial encapsulated changes of mediastinum (red arrow) revealing the possibility of mediastinal abscess, some pleural effusion (yellow arrow), and pericardial effusion (white arrow). (**C**). CT showing mediastinal abscess disappeared when the patient was discharged.

The patient experienced fever for fifteen days before he visited the pediatric infectious department of our hospital. He had initially developed a high fever of 39°C, two or three times daily, without chills, lethargy, cough or vomit. The patient was treated at home with only oral antipyretic drugs. He was admitted to the local hospital four days later as he developed a high fever of 42°C, two or three times daily, accompanied by chills, lethargy, decreased appetite, tachycardia, tachypnea, and mild cough. Physical examinations revealed enlarged tonsils with yellow discharge, 0.5 cm × 2 cm lymphadenopathy on the right side of the neck and few rales in bilateral lungs. Laboratory tests showed a normal white blood cell count (WBC, 9.82 × 10^9^/L; normal range, 4–10 × 10^9^/L) and elevated levels of C-reactive protein (CRP, 206.6 mg/L; normal range, <0.5 mg/L) and procalcitonin (PCT, 10.831 ng/ml; normal range, <0.5 ng/ml). The first sample of blood culture collected on admission was negative for bacterial growth. Radiograph of the chest revealed bilateral bronchopneumonia. The doppler echocardiography was normal and ultrasound of the neck did not find any obvious mass or liquid anechoic area. Even after intravenous administration of cefuroxime (25 mg/kg, q6h) for two days and cefoperazone sodium and sulbactam sodium (20 mg/kg, q6h) for three days (Table 1), the patient's fever persisted. Subsequently, the second sample of blood culture, collected five days after admission, revealed the growth of *S. aureus* which was demonstrated to be methicillin-susceptible. Computed tomography (CT) of the chest showed bilateral scattered infiltrates in lungs (Figure 1A), an enlarged mediastinum, some pleural effusion, and pericardial effusion (Figure 1B). Moreover, the WBC count and CRP levels increased to 14 × 10^9^/L and 295.1 mg/L, respectively. Physicians considered the occurrence of SAB and modified the antibiotic treatment to intravenous vancomycin (50 mg/kg, q6h) after excluding central nervous infection. The symptoms alleviated after four days of intravenous vancomycin therapy. Nine days after admission to the local hospital, contrast-enhanced CT of the chest revealed scattered infiltrates in the bilateral lungs (Figure 2A) and encapsulated MA (Figure 2B), and the patient was transferred to the pediatric surgery unit of another hospital for abscess incision and drainage.

**Table 1:** Clinical course of the patient.

Hospitals	Day	Antibiotic	Dose	Clinical course
Hospital 1	Day 1-2	cefuroxime	25 mg/kg, q6h	Fever with lethargy, tachycardia, tachypnea, elevated CRP and PCT.
Hospital 1	Day 3-5	cefoperazone sodium and sulbactam sodium	20 mg/kg, q6h	Persistence of symptoms, an enlarged mediastinum, increase of CRP
Hospital 1	Day 6-9	vancomycin	10 mg/kg, q6h	Detection of MSSA, discovery of MA, improvement of symptoms
Hospital 2	Day 10-21	Vancomycin oxacillin	10 mg/kg, q6h 50 mg/kg, q6h	Fever subsidence, decrease of CRP, partial absorption of MA
Hospital 3	Day 22-35	oxacillin	50 mg/kg, q6h	Recovery

MA, mediastinal abscess; MSSA, methicillin-susceptible *Staphylococcus aureus*.

The patient received intravenous oxacillin (50 mg/kg, q6h) and vancomycin (10 mg/kg, q6h) treatment in the surgical ward before surgery. With this therapy regimen, the symptoms improved gradually and contrast-enhanced CT of the chest obtained after eleven days suggested partial absorption of MA. Afterwards, the patient was transferred to our hospital for continuous conservation treatment.

Physical examination of the patient at our hospital on admission revealed the vital signs were normal. He had pharyngeal hyperemia, with bilateral tonsil hypertrophy without exudate. The rest of the physical parameters were normal. Laboratory tests showed that the WBC count was 6.6 × 10^9^/L and the CRP level was less than 0.5 mg/L. Tests for immune function and tuberculosis yielded normal results.

The patient was diagnosed to be septic, by methicillin-susceptible *S. aureus* (MSSA) and non-traumatic MA. After fourteen consecutive days of intravenous oxacillin (50 mg/kg, q6h) treatment, the blood culture was negative for MSSA twice, and MA disappeared (Figure 2C). The patient fully recovered and was discharged. He did not show any untoward symptom during the one-month follow-up.

## Discussion and conclusions

*Staphylococcus aureus* is one of the leading pathogens causing community-acquired and hospital-acquired bacteremia. Most children diagnosed with SAB manifest symptoms such as local infective lesions related to osteomyelitis, arthritis, skin and soft tissue infections, pneumonia, and intravascular catheter infections. The ratio of patients diagnosed with SAB without focused infection only accounts for 5%–7% (5, 11). However, in this case, the patient showed no signs of local infective lesions. The respiratory system manifestations, including a mild cough, few fixed rales, and scattered infiltrates in the bilateral lungs could not be correlated with *S. aureus* pneumonia. Therefore, the origin of SAB was unclear.

Non-traumatic MA, which can be caused by hematogenous or lymphatic spread from distant sites of infection, or direct extension along contiguous anatomic pathways and fascial planes such as peritonsillar or retropharyngeal abscess (12), odontogenic infection, mediastinal lymph node tuberculous abscess (13) and *S. aureus* pneumonitis (14), is rare in children. The most common etiology identified in non-traumatic mediastinitis is *S. aureus* (9, 10), manifesting as disseminated staphylococcal infection (15). Non-traumatic MA related to SAB was uncommon among the pediatric population. Smith et al. reported a case that SAB originating from septic arthritis lead to an anterior mediastinal abscess in a 11-year-old boy who recovered by surgical drainage and antibiotic therapy (16). In this case, the patient had no surgeries, wounds or clues of descending infection. As lack of symptoms including swallowing disorders, pain, or impaired movement of the neck, the clinical evidence for pharyngeal or cervical infection is insufficient despite the patient had tonsil hypertrophy and lymphadenopathy on the right side of neck. In addition, the ultrasound of the neck did not reveal any obvious mass or liquid anechoic area. Hence, the MA was considered to be a result of SAB. After antibiotic therapy for MSSA, the MA absorbed completely.

Early diagnosis and aggressive treatments of SAB and MA are essential for decreasing mortality and morbidity. Contrast-enhanced CT scan is indispensable to confirm the presence of loculations, the extent of the MA, and its relationship with surrounding organs. Treatment of acute MA is based on the administration of antibiotics and control of the source of infection by surgical debridement (8). The time of antibiotic administration is recognized to be the main determinant of SAB outcomes (17, 18). Empirical antibiotic treatment for children with suspected SAB depends on comprehensive considerations including the source of infection, severity, community-related or hospital-related origin, and the prevalence of methicillin-resistant *S. aureus* (MRSA) in the community. For children with life-threatening infection with suspected SAB, empirical therapy consists of vancomycin plus nafcillin/oxacillin (19, 20), which can cover both MRSA and MSSA in most cases. Vancomycin is the primary choice for hospital-related *S. aureus* infection and community-acquired MRSA infection. However, once *S. aureus* is confirmed to be methicillin-susceptible, the antimicrobial regimen should be modified (16). In this case, the patient with SAB received empirical vancomycin treatment in order to cover MRSA. Despite MSSA, oxacillin and vancomycin were applied in the surgical unit, probably in consideration of severe infection or insufficient experience in treating children diagnosed with SAB complicated with non-traumatic mediastinal abscess. The antibiotic was adjusted to oxacillin based on the drug susceptibility of *S. aureus* when the patient was transferred to the pediatric infection department of our hospital. Immediate antibiotic treatment and surgical drainage are both indispensable for MA. Sanchez et al. reported a severe case of community-acquired MRSA pericarditis with extension to the mediastinum and carotid sheath in a previously healthy 8-month-old infant who was successfully treated with surgical interventions and antibiotics (9). Lira et al. also reported a case of mediastinitis in a five-year-old child, successfully treated with 4 weeks of intravenous antibiotics. The patient in the case also recovered well without surgical involvement. The potential reason was unclear. Maybe it was related to prompt initiation of antibiotic treatment and appropriate duration.

In conclusion, physicians should pay attention to MA among children with SAB. Early diagnosis and treatment were essential to acquiring a satisfying outcome. Some patients can recover well without surgery.

## Data Availability

The original contributions presented in the study are included in the article/Supplementary Material, further inquiries can be directed to the corresponding author/s.
